# Supplementary material for: Sex Differences in Cochlear Transcriptomes in Horseshoe Bats
Source: Animals (Basel). 2024 Apr 14;14(8):1177. doi: 10.3390/ani14081177 (PMC11047584; doi:10.3390/ani14081177)
Supplement: Supplementary file 1 [file animals-14-01177-s001.zip › Figure S4.pdf]

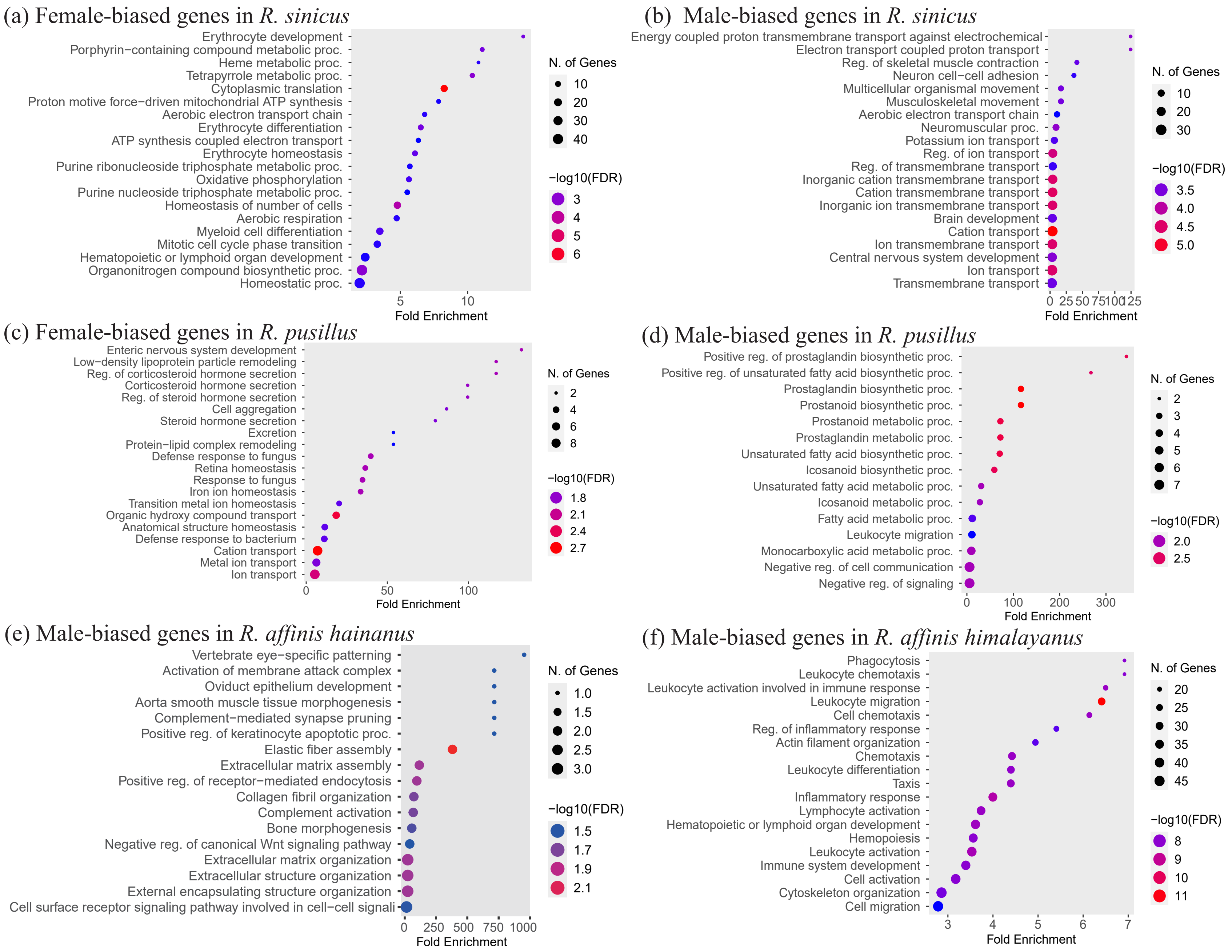
(a) Female-biased genes in R. sinicus
Erythrocyte development
Porphyrin−containing compound metabolic proc.
Heme metabolic proc.
Tetrapyrrole metabolic proc.
Cytoplasmic translation
Proton motive force−driven mitochondrial ATP synthesis
Aerobic electron transport chain
Erythrocyte differentiation
ATP synthesis coupled electron transport
Erythrocyte homeostasis
Purine ribonucleoside triphosphate metabolic proc.
Oxidative phosphorylation
Purine nucleoside triphosphate metabolic proc.
Homeostasis of number of cells
Aerobic respiration
Myeloid cell differentiation
Mitotic cell cycle phase transition
Hematopoietic or lymphoid organ development
Organonitrogen compound biosynthetic proc.
Homeostatic proc.
5
10
Fold Enrichment
N. of Genes
10
20
30
40
−log10(FDR)
3
4
5
6
(b) Male-biased genes in R. sinicus
Energy coupled proton transmembrane transport against electrochemical
Electron transport coupled proton transport
Reg. of skeletal muscle contraction
Neuron cell−cell adhesion
Multicellular organismal movement
Musculoskeletal movement
Aerobic electron transport chain
Neuromuscular proc.
Potassium ion transport
Reg. of ion transport
Reg. of transmembrane transport
Inorganic cation transmembrane transport
Cation transmembrane transport
Inorganic ion transmembrane transport
Brain development
Cation transport
Ion transmembrane transport
Central nervous system development
Ion transport
Transmembrane transport
0
25
50
75
100
125
Fold Enrichment
N. of Genes
10
20
30
−log10(FDR)
3.5
4.0
4.5
5.0
(c) Female-biased genes in R. pusillus
Enteric nervous system development
Low−density lipoprotein particle remodeling
Reg. of corticosteroid hormone secretion
Corticosteroid hormone secretion
Reg. of steroid hormone secretion
Cell aggregation
Steroid hormone secretion
Excretion
Protein−lipid complex remodeling
Defense response to fungus
Retina homeostasis
Response to fungus
Iron ion homeostasis
Transition metal ion homeostasis
Organic hydroxy compound transport
Anatomical structure homeostasis
Defense response to bacterium
Cation transport
Metal ion transport
Ion transport
0
50
100
Fold Enrichment
N. of Genes
2
4
6
8
−log10(FDR)
1.8
2.1
2.4
2.7
(d) Male-biased genes in R. pusillus
Positive reg. of prostaglandin biosynthetic proc.
Positive reg. of unsaturated fatty acid biosynthetic proc.
Prostaglandin biosynthetic proc.
Prostanoid biosynthetic proc.
Prostanoid metabolic proc.
Prostaglandin metabolic proc.
Unsaturated fatty acid biosynthetic proc.
Icosanoid biosynthetic proc.
Unsaturated fatty acid metabolic proc.
Icosanoid metabolic proc.
Fatty acid metabolic proc.
Leukocyte migration
Monocarboxylic acid metabolic proc.
Negative reg. of cell communication
Negative reg. of signaling
0
100
200
300
Fold Enrichment
N. of Genes
2
3
4
5
6
7
−log10(FDR)
2.0
2.5
(e) Male-biased genes in R. affinis hainanus
Vertebrate eye−specific patterning
Activation of membrane attack complex
Oviduct epithelium development
Aorta smooth muscle tissue morphogenesis
Complement−mediated synapse pruning
Positive reg. of keratinocyte apoptotic proc.
Elastic fiber assembly
Extracellular matrix assembly
Positive reg. of receptor−mediated endocytosis
Collagen fibril organization
Complement activation
Bone morphogenesis
Negative reg. of canonical Wnt signaling pathway
Extracellular matrix organization
Extracellular structure organization
External encapsulating structure organization
Cell surface receptor signaling pathway involved in cell−cell signali
0
250
500
750
1000
Fold Enrichment
N. of Genes
1.0
1.5
2.0
2.5
3.0
−log10(FDR)
1.5
1.7
1.9
2.1
(f) Male-biased genes in R. affinis himalayanus
Phagocytosis
Leukocyte chemotaxis
Leukocyte activation involved in immune response
Leukocyte migration
Cell chemotaxis
Reg. of inflammatory response
Actin filament organization
Chemotaxis
Leukocyte differentiation
Taxis
Inflammatory response
Lymphocyte activation
Hematopoietic or lymphoid organ development
Hemopoiesis
Leukocyte activation
Immune system development
Cell activation
Cytoskeleton organization
Cell migration
3
4
5
6
7
Fold Enrichment
N. of Genes
20
25
30
35
40
45
−log10(FDR)
8
9
10
11
