# Supplementary figures and images for: Sex Differences in Cochlear Transcriptomes in Horseshoe Bats
Source: Animals (Basel). 2024 Apr 14;14(8):1177. doi: 10.3390/ani14081177 (PMC11047584; doi:10.3390/ani14081177)

(a) *R. sinicus*

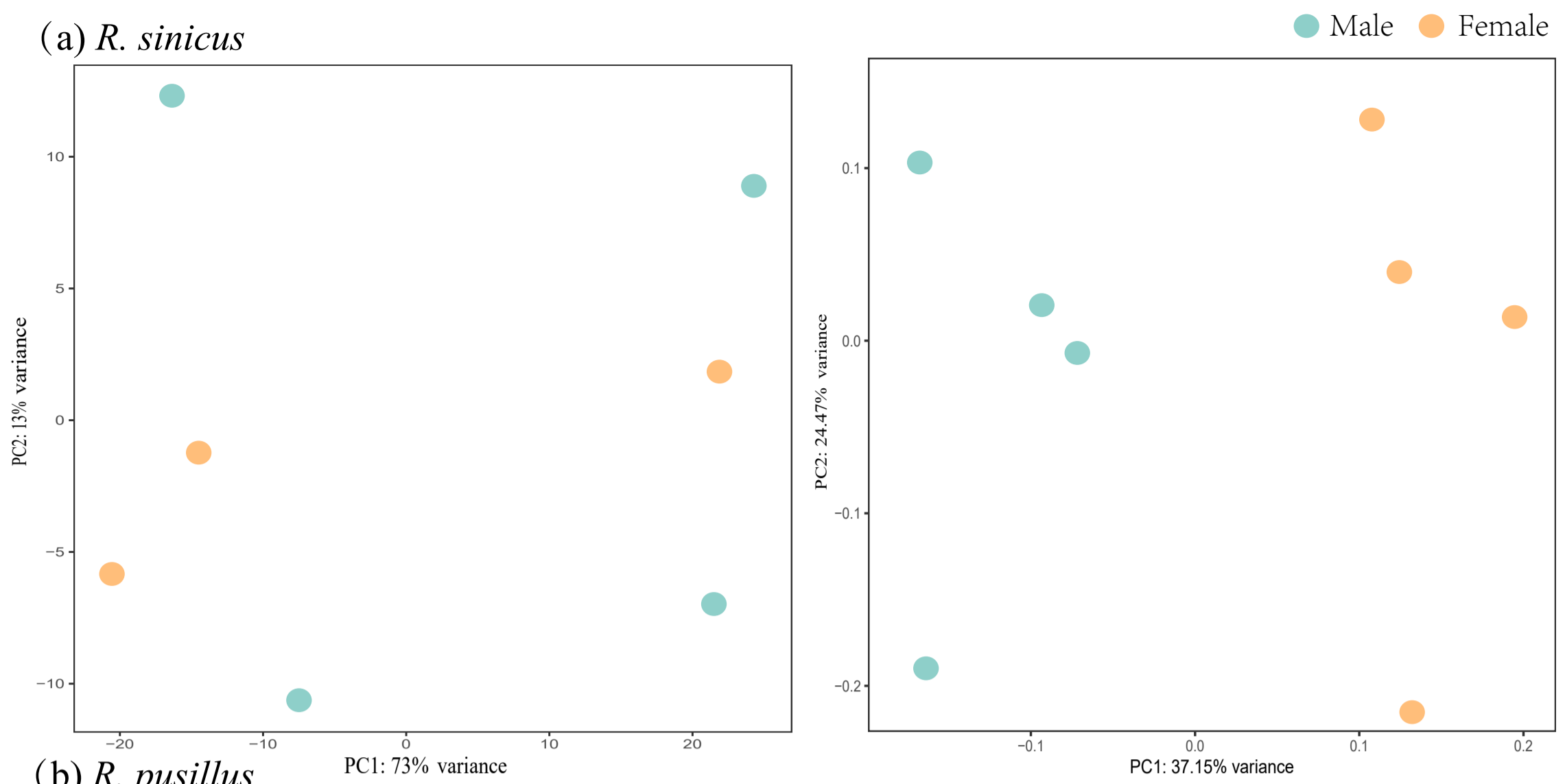

(b) *R. pusillus*

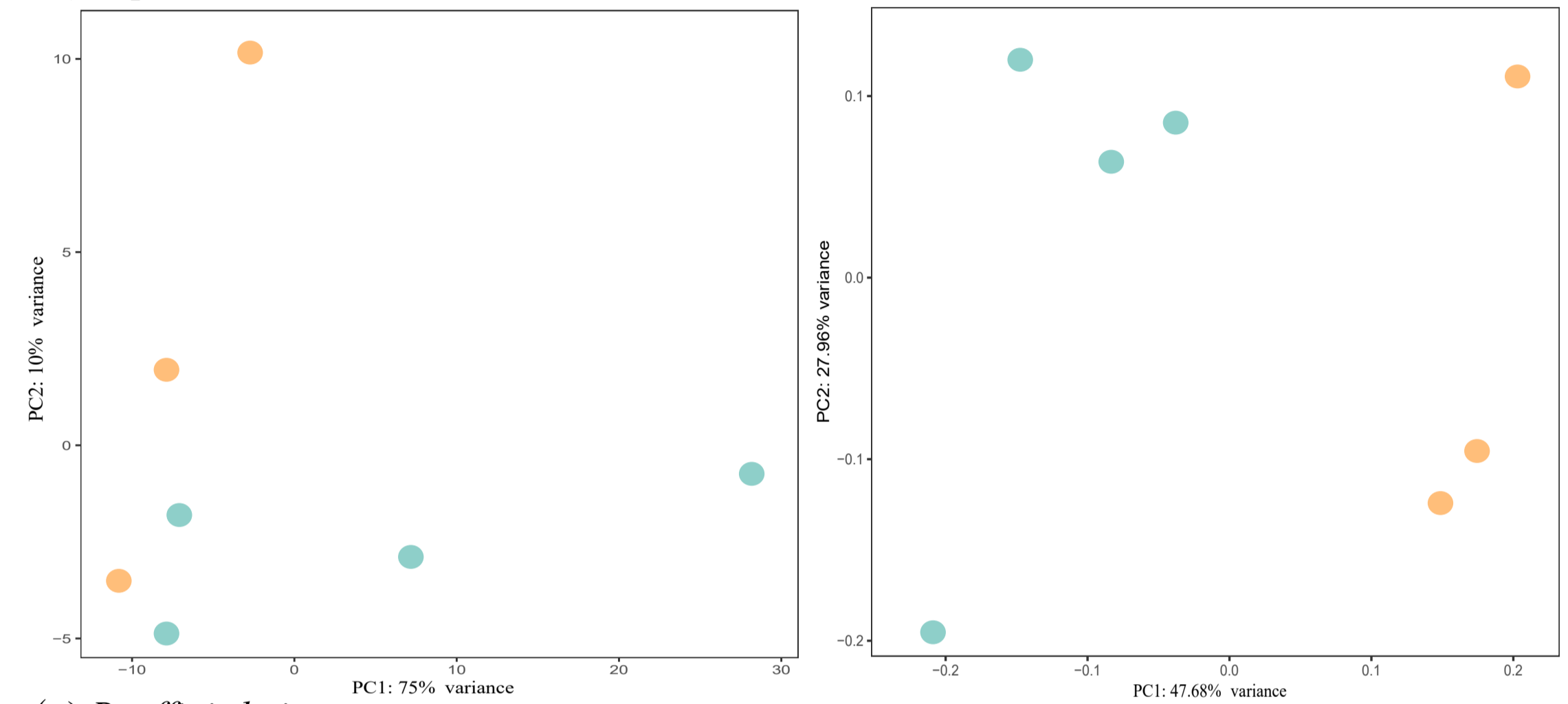

(c) *R. affinis hainanus*

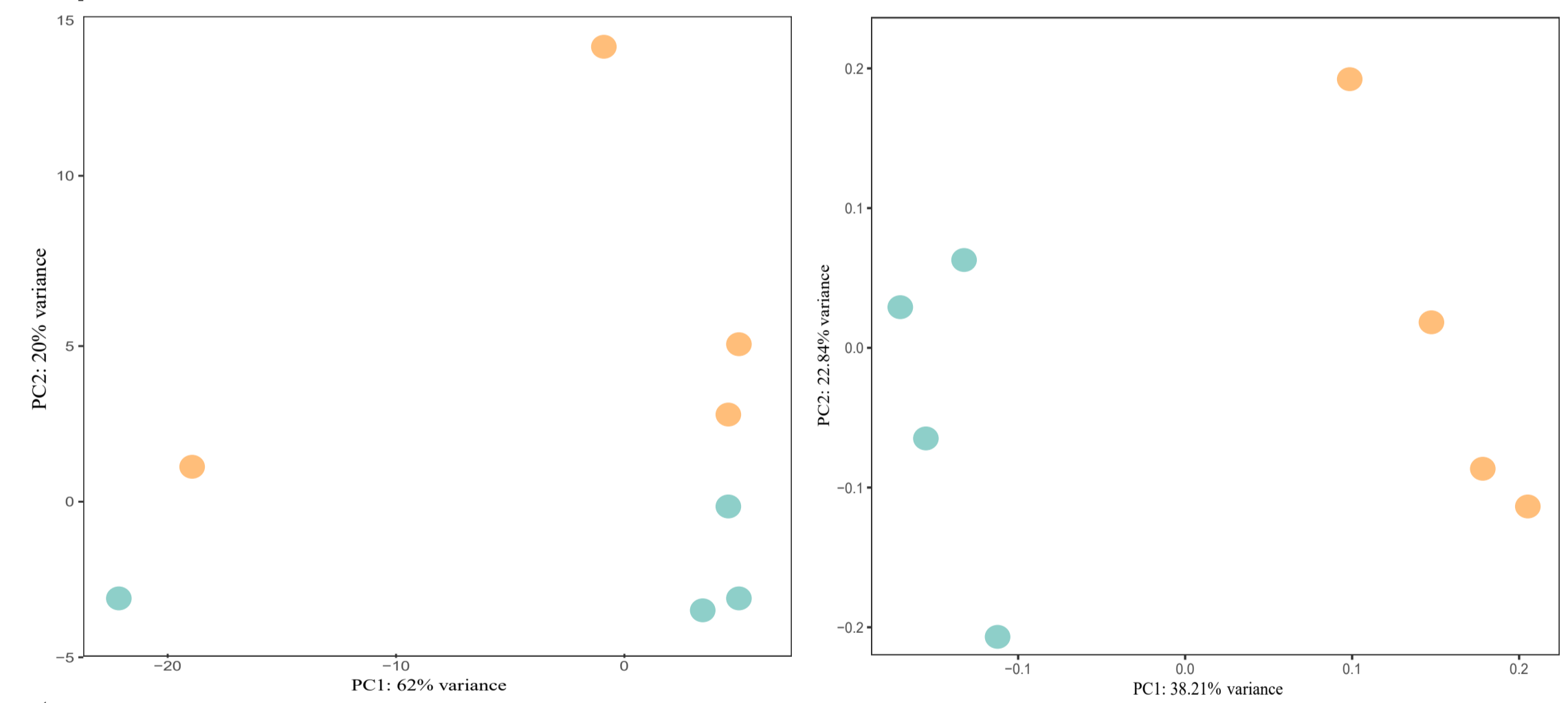

(d) *R. affinis himalayanus*

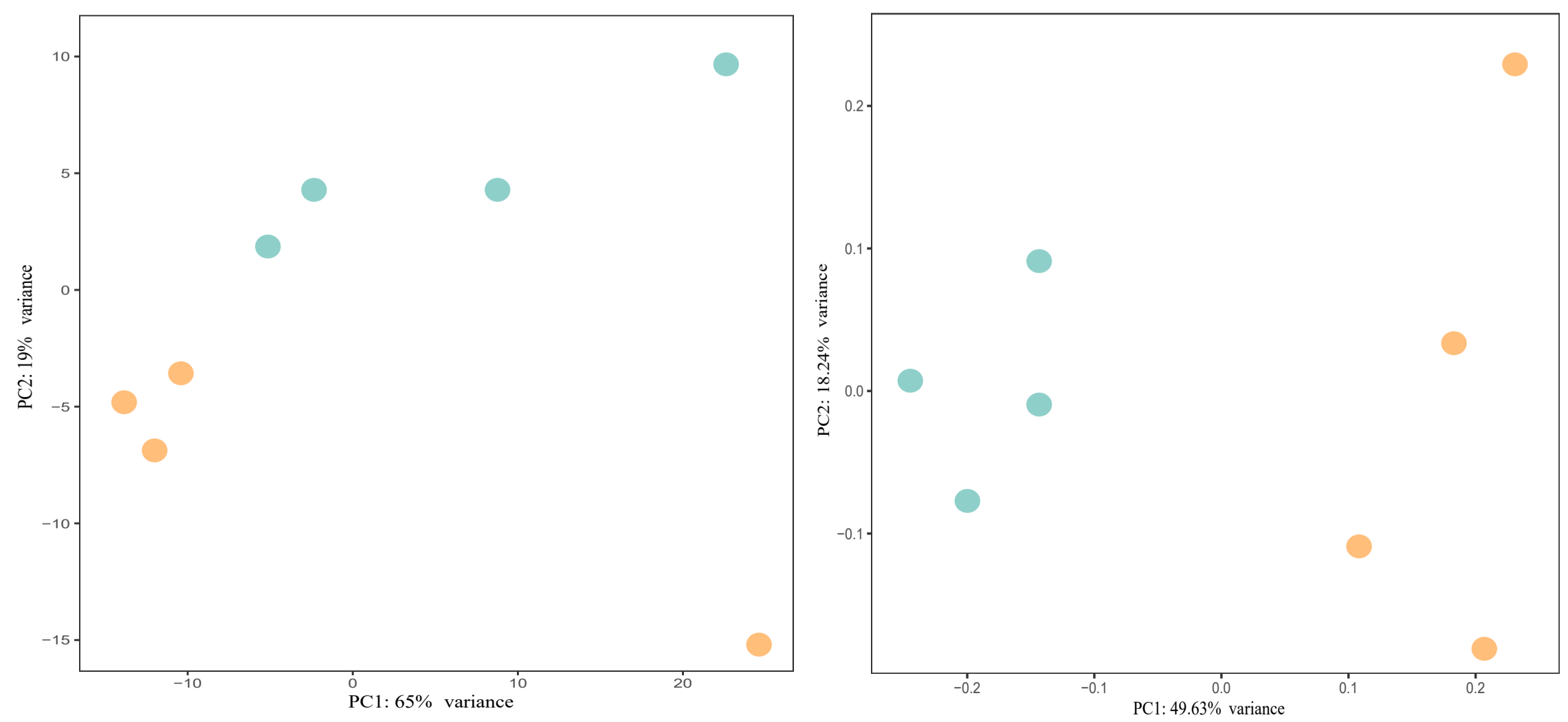

Supplement: Supplementary file 1 [file animals-14-01177-s001.zip › Figure S1.pdf]

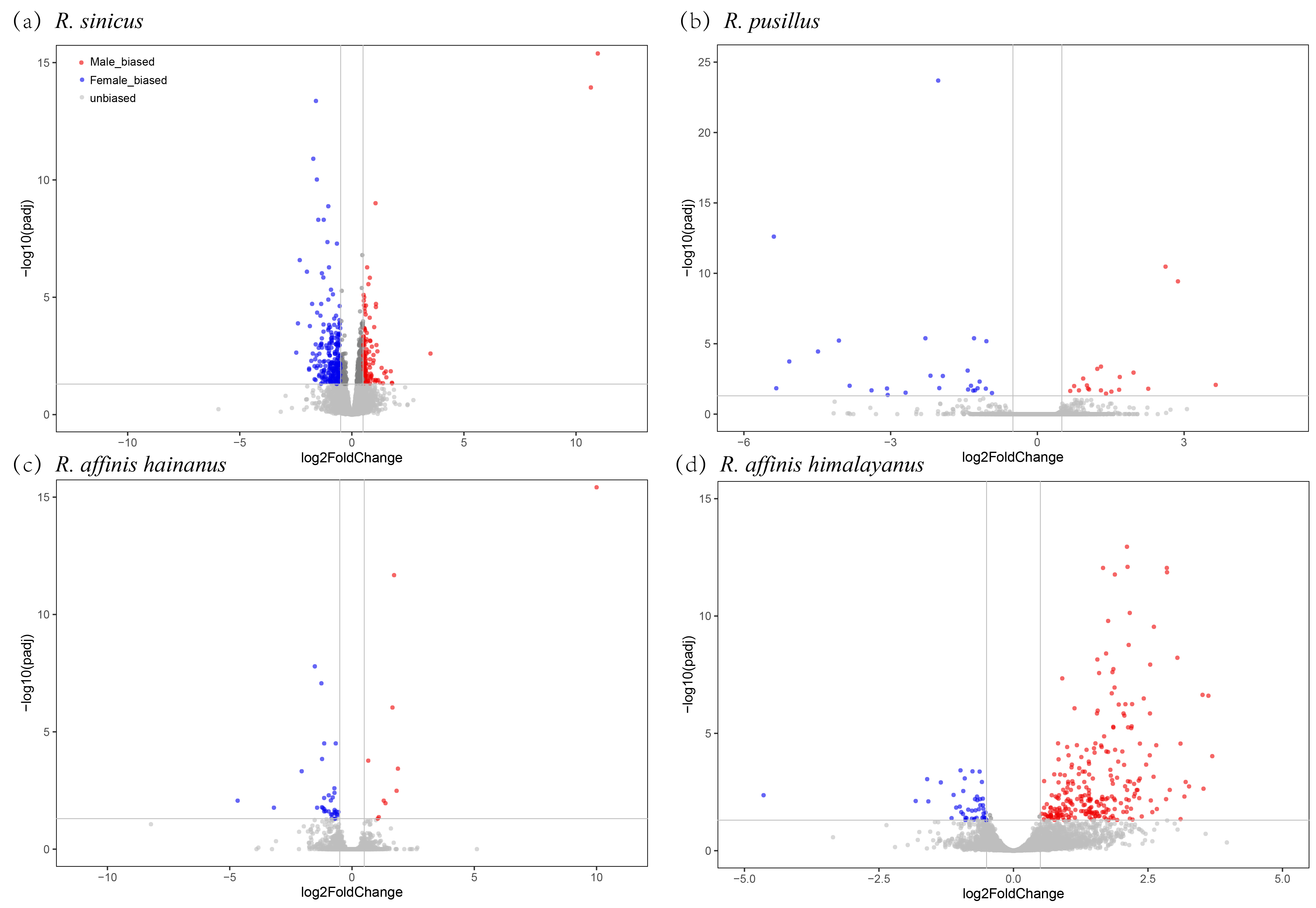

Supplement: Supplementary file 1 [file animals-14-01177-s001.zip › Figure S2.jpg]

(a) *R. sinicus*

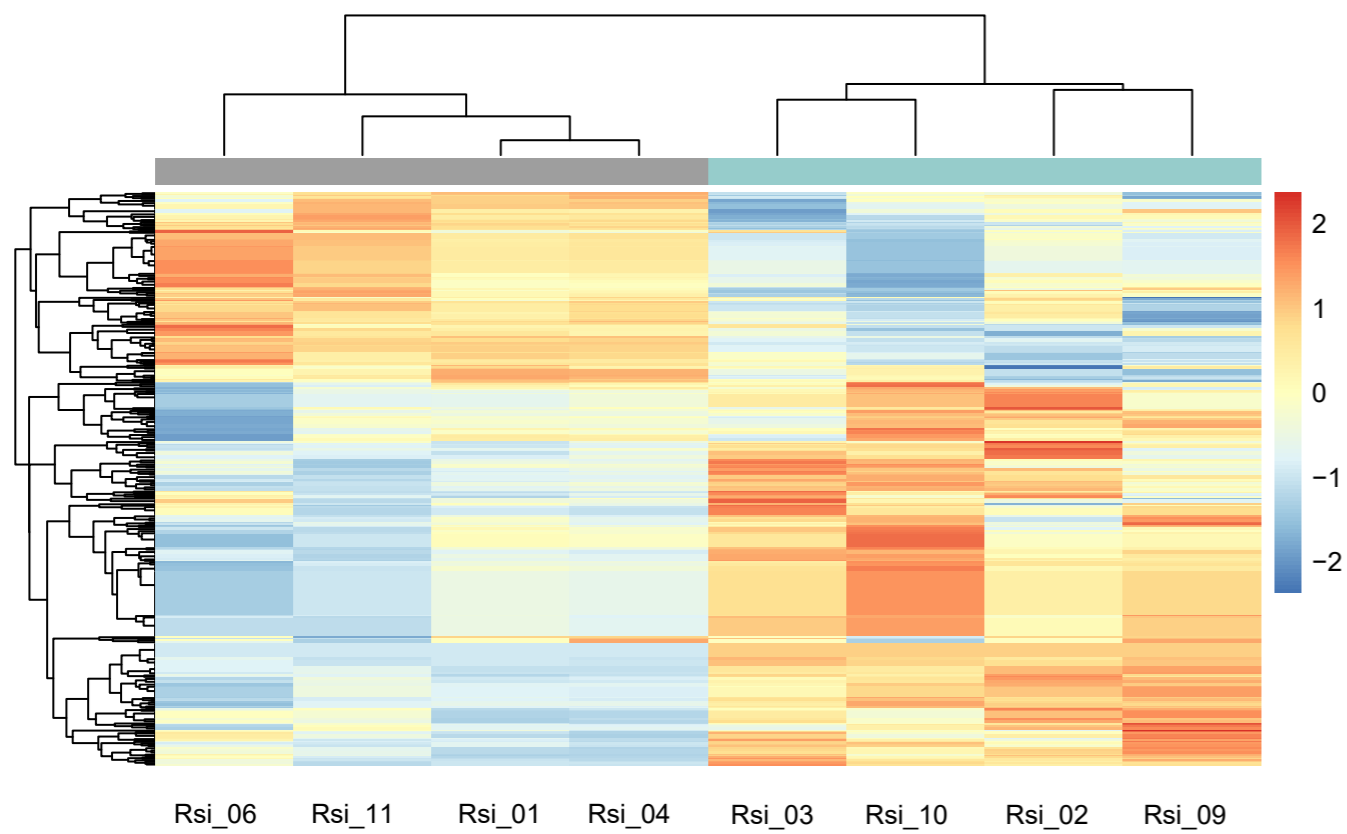

(b) *R. pusillus*

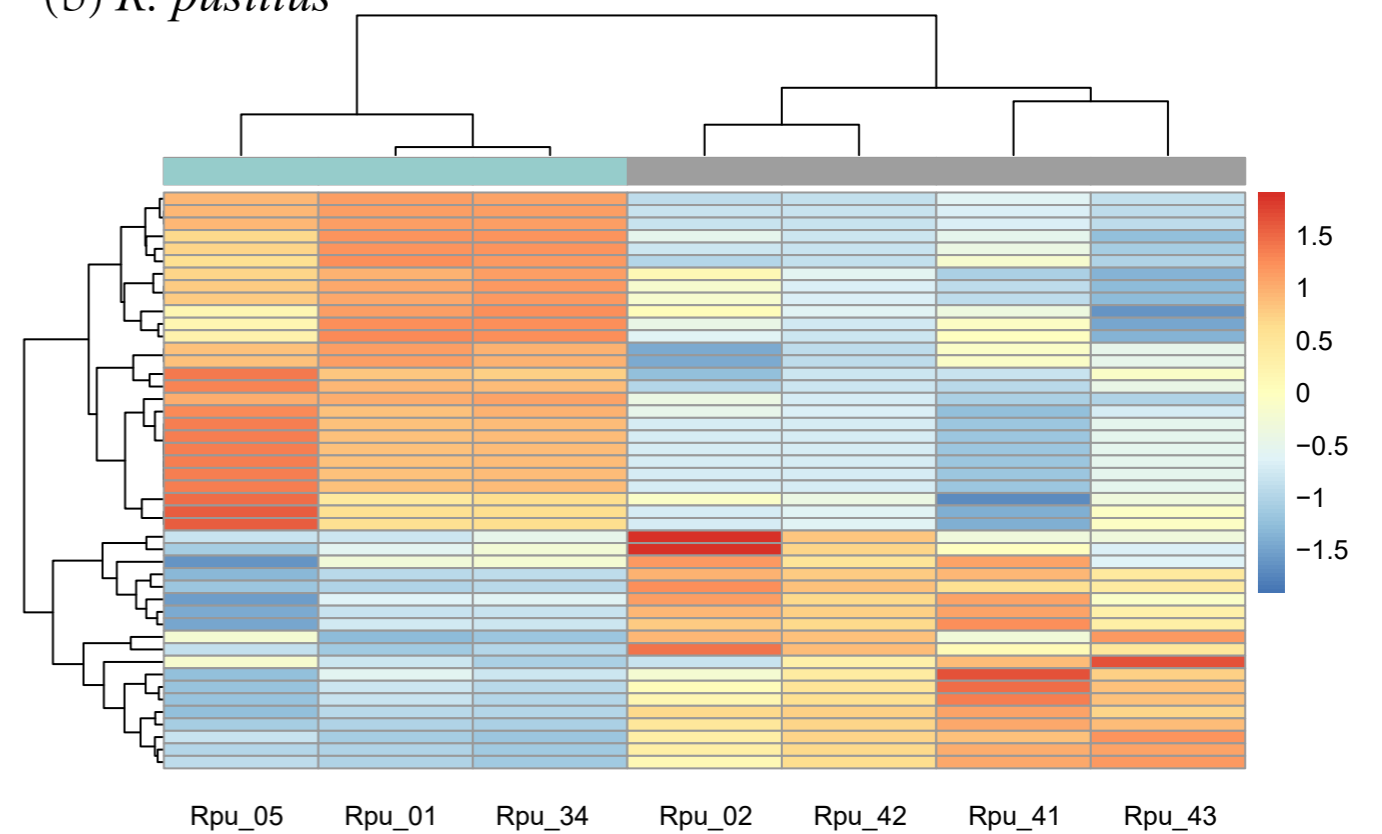

(c) *R. affinis hainanus*

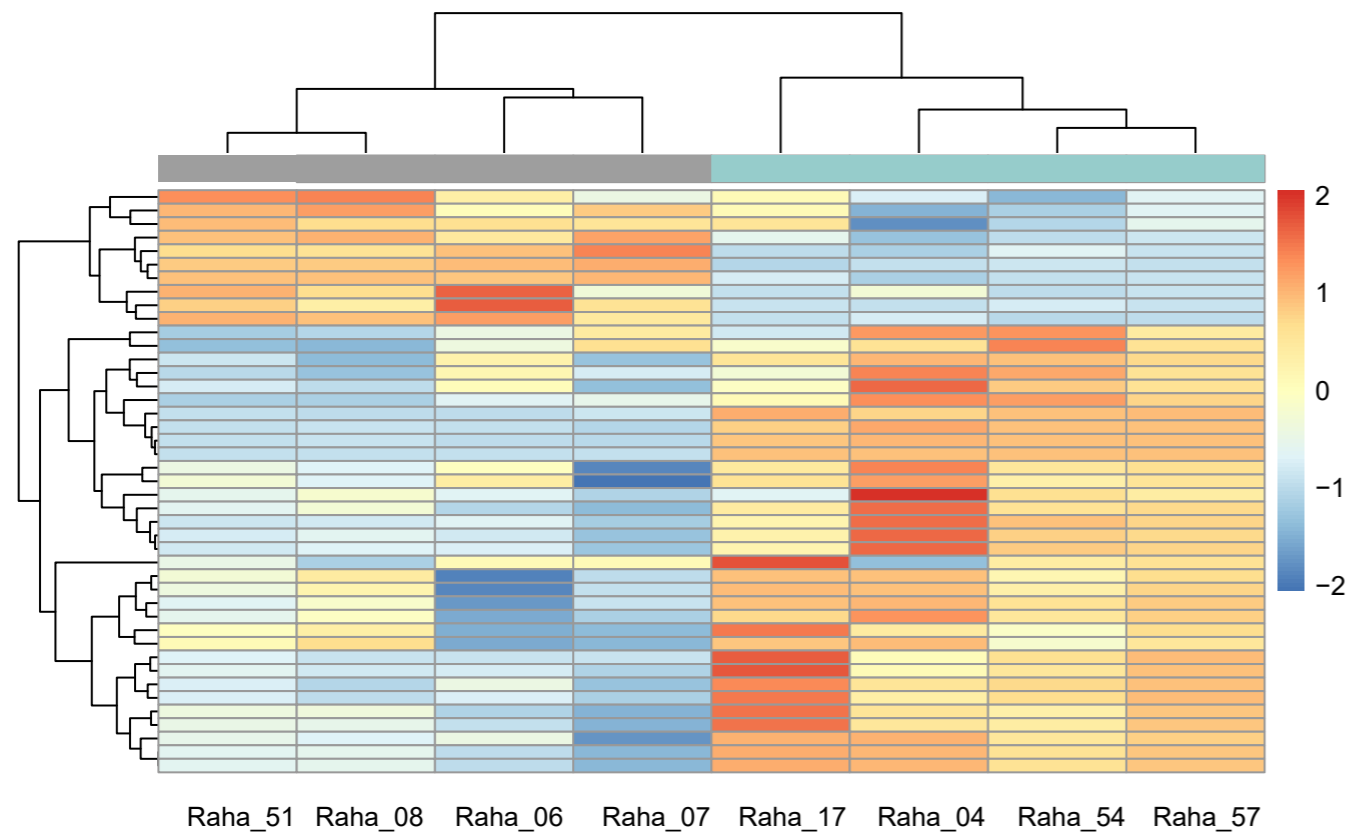

(d) *R. affinis himalayanus*

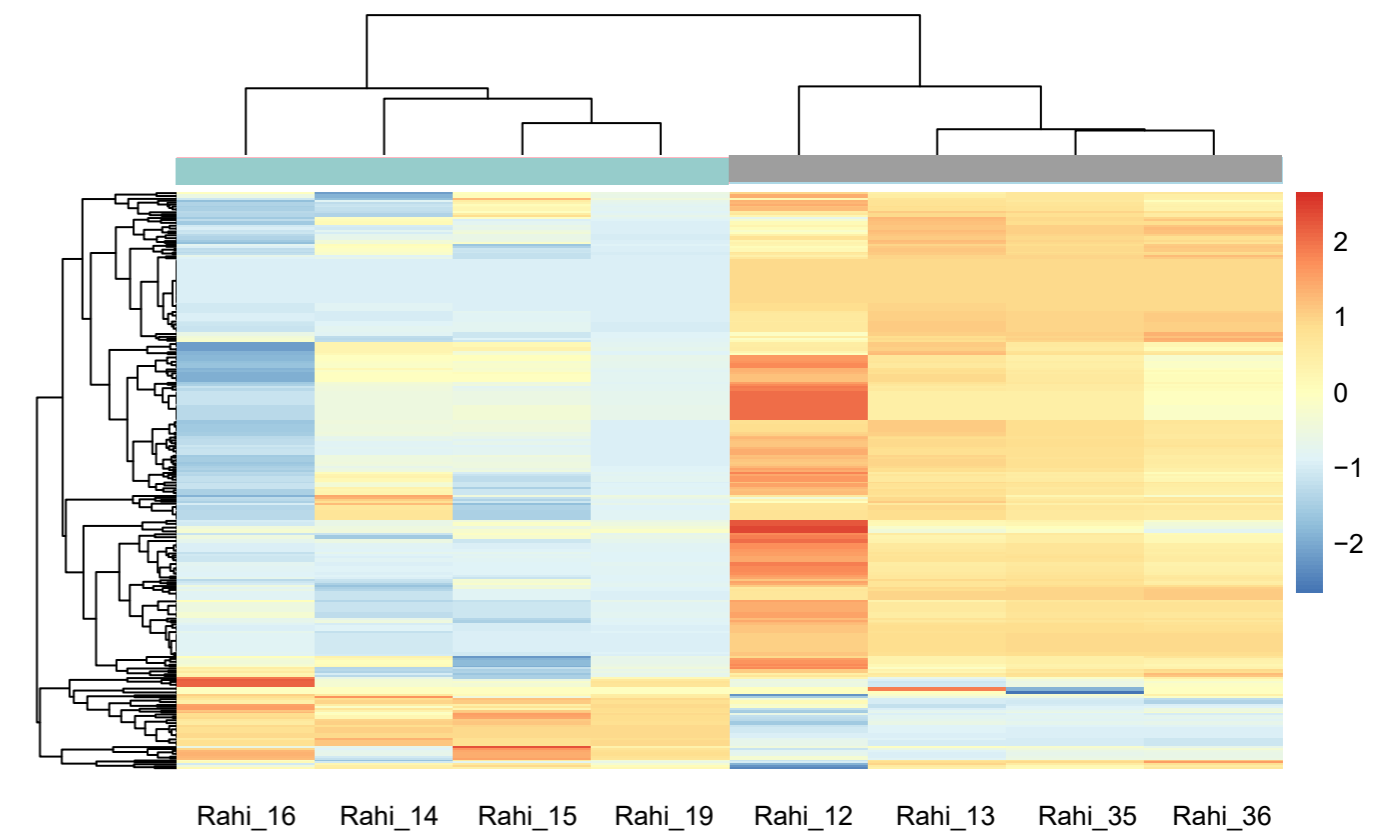

Supplement: Supplementary file 1 [file animals-14-01177-s001.zip › Figure S3.pdf]

Male\_biased

Female\_biased

394

22

347

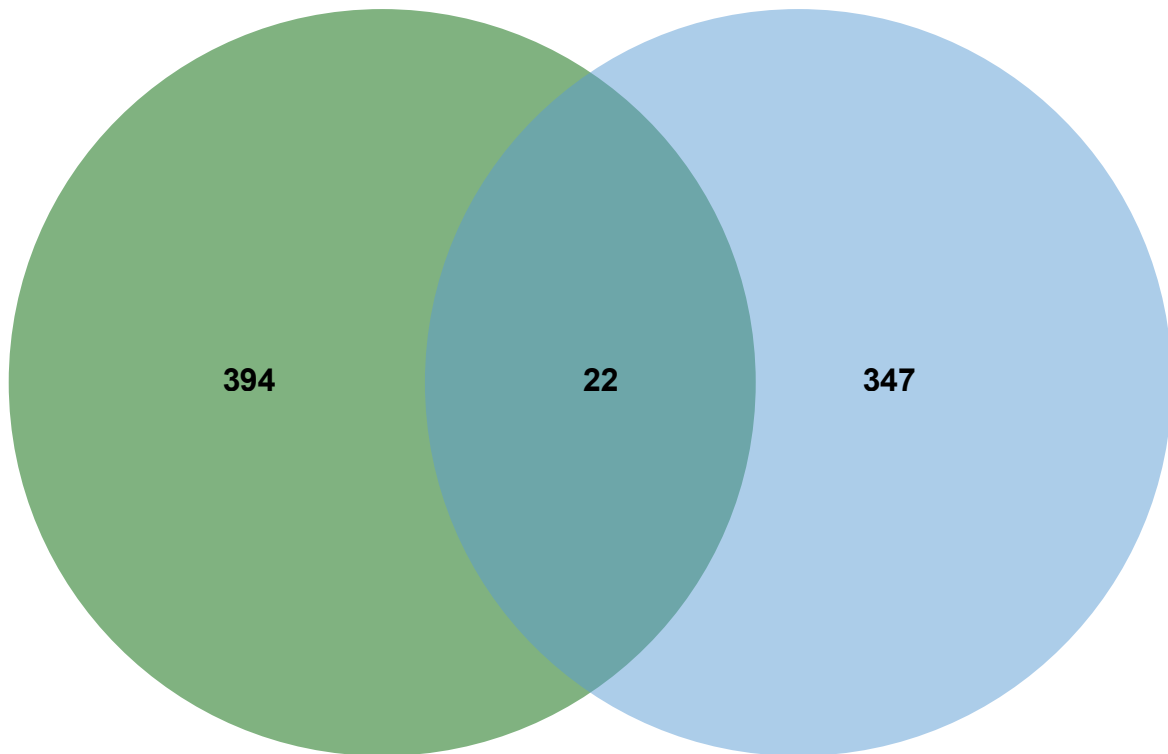

Supplement: Supplementary file 1 [file animals-14-01177-s001.zip › Figure S5.pdf]

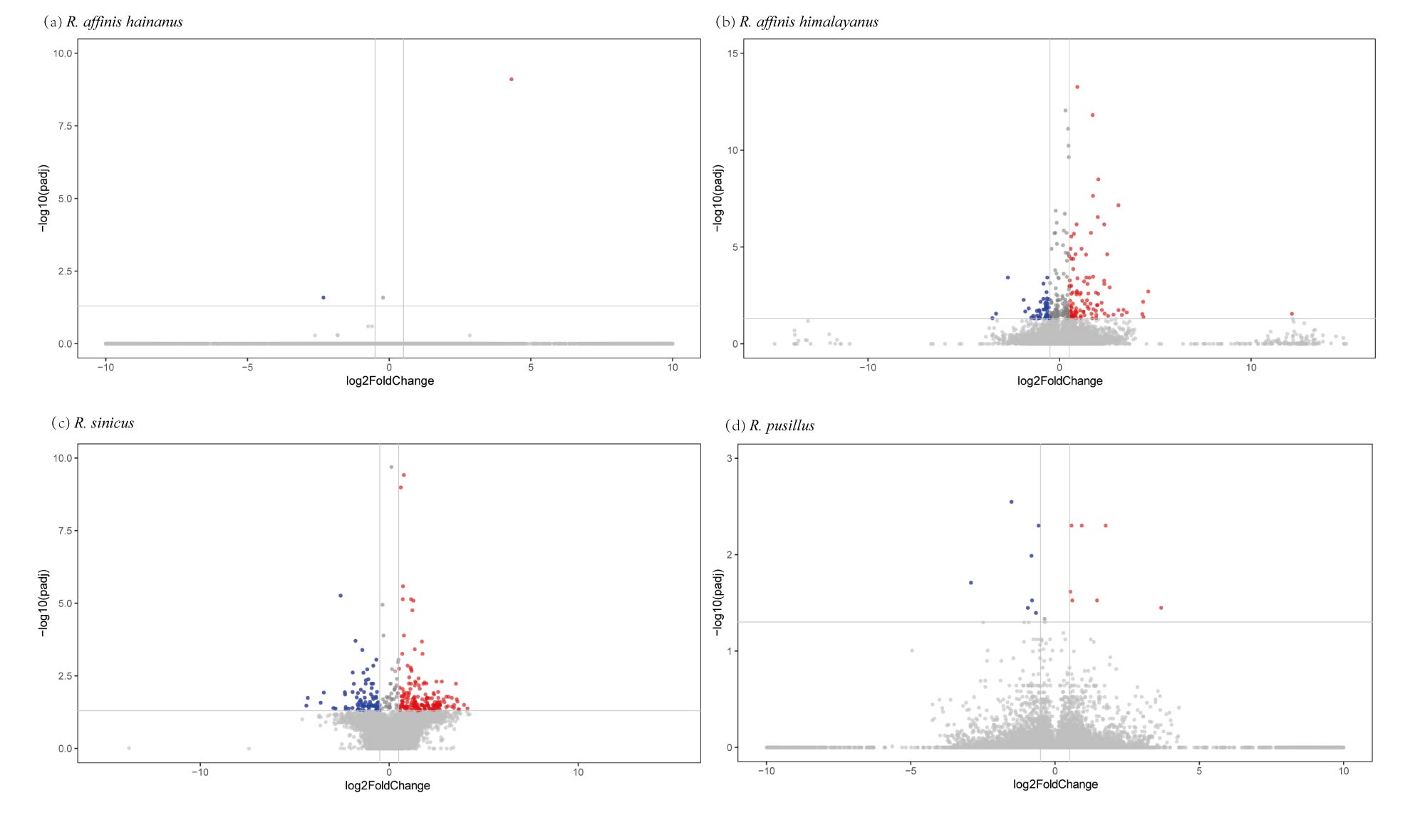

Supplement: Supplementary file 1 [file animals-14-01177-s001.zip › Figure S6.jpg]

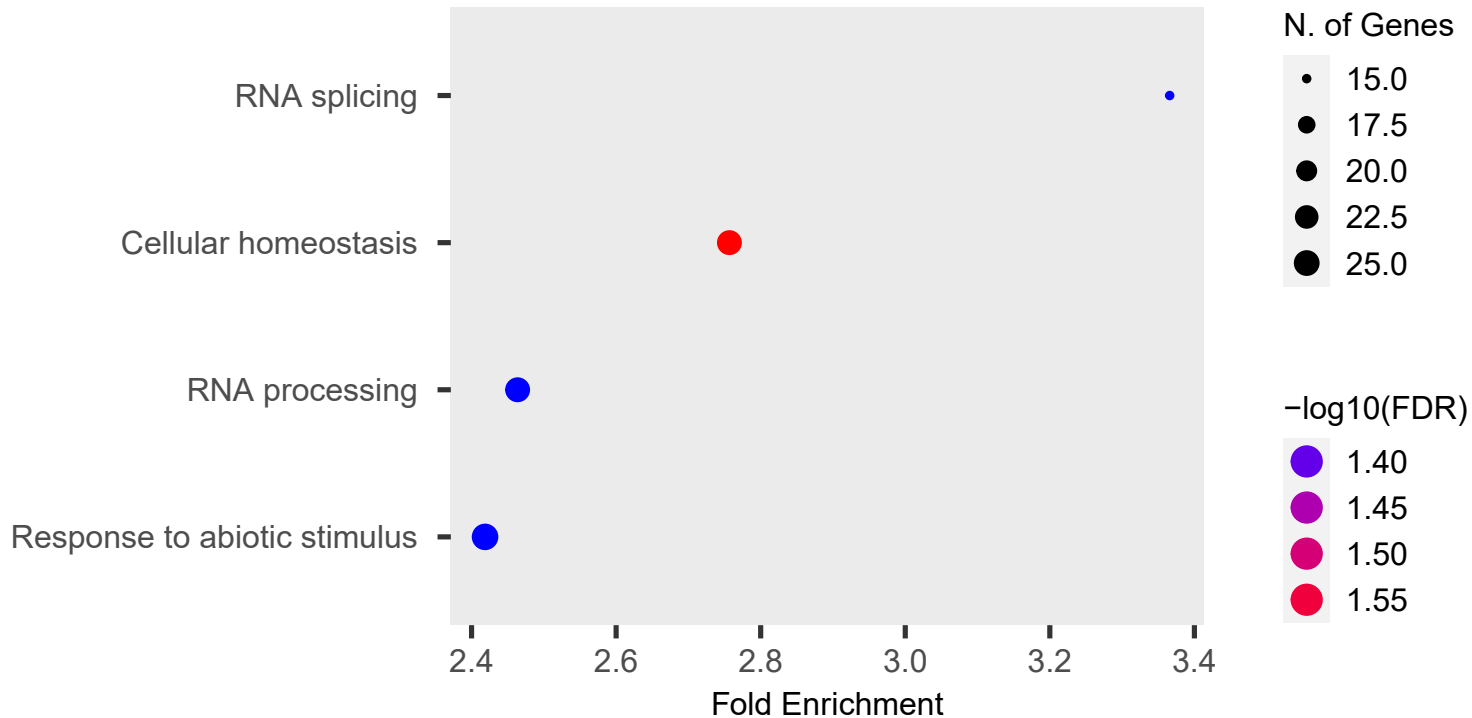

Supplement: Supplementary file 1 [file animals-14-01177-s001.zip › Figure S7.pdf]
